# Optimized In‐Solution and Gas‐Phase Chemistry Enables High‐Efficiency Interactome Mapping by DSBSO‐Based Cross‐Linking Mass Spectrometry

**DOI:** 10.1002/anie.202518355

**Published:** 2025-11-20

**Authors:** Pin‐Lian Jiang, Ying Zhu, Jiaxin Cai, Cong Wang, Mei Wu, Ke Pu, Fan Liu

**Affiliations:** ^1^ Department of Structural Biology Leibniz‐Forschungsinstitut für Molekulare Pharmakologie (FMP) Berlin Germany; ^2^ Institute of Chemistry and Biochemistry Freie Universität Berlin Berlin Germany; ^3^ Absea Biotechnology Ltd Berlin Germany; ^4^ Charité Universitätsmedizin Berlin Berlin Germany

**Keywords:** Cross‐linking mass spectrometry, Protein‐protein interactions, Proteomics

## Abstract

Cross‐linking mass spectrometry (XL–MS) allows characterizing protein structures and interactions in highly complex samples. The enrichable disuccinimidyl bissulfoxide (DSBSO) cross‐linker has enabled comprehensive XL–MS studies of human cells. However, existing DSBSO workflows demand multi‐day sample preparation with high input requirements and provide insufficient detection sensitivity. Here, we systematically optimize the in solution and gas‐phase chemistry of azide‐A‐DSBSO‐based XL–MS. Importantly, we reduce sample preparation time to 10 h and introduce StageTip‐based strong cation exchange (SCX) separation to concomitantly remove nonvolatile salts and interfering contaminants. Applying our streamlined SCX protocol to intact *Bacillus subtilis* reduced sample consumption 15‐fold compared to conventional size‐exclusion chromatography‐based azide‐A‐DSBSO XL‐MS and doubled the identification numbers, yielding 3,209 protein interactions at a 1% false‐discovery rate. These results illustrate that our optimized workflow unites speed, analytical depth, and resource efficiency, making XL–MS amenable to high‐throughput interactome profiling of complex biological samples.

## Graphical abstract

We developed a rapid, high‐throughput XL–MS workflow using the enrichable azide‐A‐DSBSO cross‐linker and StageTip‐based SCX fractionation. It drastically reduces preparation time and enhances sensitivity, providing a scalable platform for high‐throughput profiling of protein interaction networks.

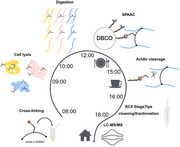

## Introduction

Cross‐linking mass spectrometry (XL–MS) is an attractive chemical strategy to characterize protein structures and interactions in a variety of systems, ranging from purified protein complexes to intact human cells.^[^ ^1^, ^2^ ^]^ In XL‐MS, proteins are covalently connected by chemical cross‐linkers that contain a short (∼10 Å) spacer arm. The cross‐linked proteins are enzymatically digested into peptides and analyzed by liquid chromatography‐mass spectrometry (LC‐MS) to localize the cross‐linked amino acids. These cross‐links impose distance constraints on protein assemblies, equal to the lengths of the cross‐linker spacer arm, and the side chains of the linked amino acids.

Cross‐linking mass spectrometry (XL‐MS) is uniquely suited for simultaneous probing of protein structures and interactions in complex biological environments, including virions^[^ ^3^, ^4^ ^]^ synapses,^[^ ^5^, ^6^ ^]^ organelles,^[^ ^5^, ^7^, ^8^, ^9^, ^10^ ^]^ and intact cells.^[^ ^11^, ^12^ ^]^ This approach provides proteome‐wide insights into protein interaction networks, binding interfaces, and spatial organization. Moreover, XL‐MS can capture transient interactions within their native cellular context, yielding an interactome landscape that is challenging to obtain by other methods.^[^ ^13^ ^]^

Compared to traditional LC‐MS‐based proteomics, a main shortcoming of XL‐MS remains its low sensitivity. Proteomics targets linear peptides, which represent the majority of species in any protein digest. XL‐MS, however, targets cross‐linked peptides, which constitute only ∼2% of the digestion mixture after protein cross‐linking.^[^ ^14^ ^]^ To address this limitation, enrichable cross‐linkers have been developed to enable the selective isolation of cross‐linked species. For example, PhoX^[^ ^14^ ^]^ and tBu‐PhoX^[^ ^12^ ^]^ cross‐linkers incorporate a phosphonate handle that can be isolated using TiO_2_ or immobilized metal ion affinity chromatography. Other enrichable cross‐linkers rely on biotin‐streptavidin affinity purifications^[^ ^15^, ^16^ ^]^ or antibody pull‐downs. ^[^ ^17^ ^]^ A growing class of cross‐linkers contain alkyne or azide groups, which can be conjugated by click chemistry, directly or via biotin‐containing molecules, to functionalized beads.^[^ ^18^ ^]^ Release of the enriched cross‐links from the beads is done by photo‐cleavage,^[^ ^18^ ^]^ reduction,^[^ ^19^ ^]^ or acid hydrolysis.^[^ ^20^, ^21^ ^]^

Complementary to enrichability, the cleavability of cross‐linkers has proven highly beneficial for the computationally efficient and analytically sensitive identification of cross‐linked peptides against full proteome databases.^[^ ^22^, ^23^, ^24^ ^]^ Both enrichability and cleavability are combined in disuccinimidyl bis‐sulfoxide (DSBSO) reagents.^[^ ^20^, ^21^ ^]^ They contain an alkyne or azide group for click chemistry‐based enrichment of the cross‐linked peptides, an acid‐cleavage site for efficient cross‐link release after enrichment, and a bis‐sulfoxide moiety cleavable by gas‐phase collisional dissociation to simplify identification of cross‐linked peptides. The alkyne variant of DSBSO (alkyne‐A‐DSBSO) has facilitated comprehensive interactome profiling, including an extensive analysis in HEK293 cells.^[^ ^11^ ^]^ While alkyne‐A‐DSBSO enrichment relies on biotinylation followed by streptavidin capture, azide‐A‐DSBSO makes the enrichment faster, and more efficiency by direct conjugation of the azide group to dibenzocyclooctyne (DBCO) beads.^[^ ^25^ ^]^ However, this procedure still requires at least three days of sample preparation and its enrichment performance has not been rigorously tested on proteome‐wide cross‐linking samples. Thus, while azide‐A‐DSBSO holds great promise for routine protein interactome mapping in highly complex biological samples, its sensitivity and speed require further investigation and optimization.

Here, we present an optimized azide‐A‐DSBSO XL‐MS protocol that reduces sample preparation time to 10 h, while enhancing enrichment efficiency and detection sensitivity. We systematically improve this workflow's in‐solution chemistry (proteolysis, DBCO enrichment, acid cleavage, strong cation exchange (SCX) fractionation) and gas‐phase chemistry (high‐field asymmetric waveform ion mobility spectrometry (FAIMS) and higher‐energy collision dissociation (HCD) parameters). Importantly, we eliminate the most prevalent contaminants of azide/DBCO‐based enrichment approaches – the + 1/+2‐charged interfering ions by cleaning up the eluted sample with StageTip‐based SCX instead of the typically employed reverse‐phase separation. Expanding StageTip‐based SCX from a single elution to eight fractions further increases protein‐protein interaction (PPI) coverage while maintaining low sample and time consumption. Applying this optimized XL‐MS protocol to 8 µg of DSBSO‐cross‐linked and enriched *Bacillus subtilis* yields 3209 PPIs, representing a > 2‐fold gain in identifications and 15‐fold reduction in sample input compared to the commonly used combination of XL‐MS and size exclusion chromatography (SEC) fractionation. This enhanced sensitivity and the rapid sample preparation provides a scalable XL‐MS platform as a foundation for future high‐throughput profiling of protein interaction networks.

## Results and Discussion

### Increasing the Efficiency of Protein Digestion and DBCO‐based Enrichment

**Figure 1:**
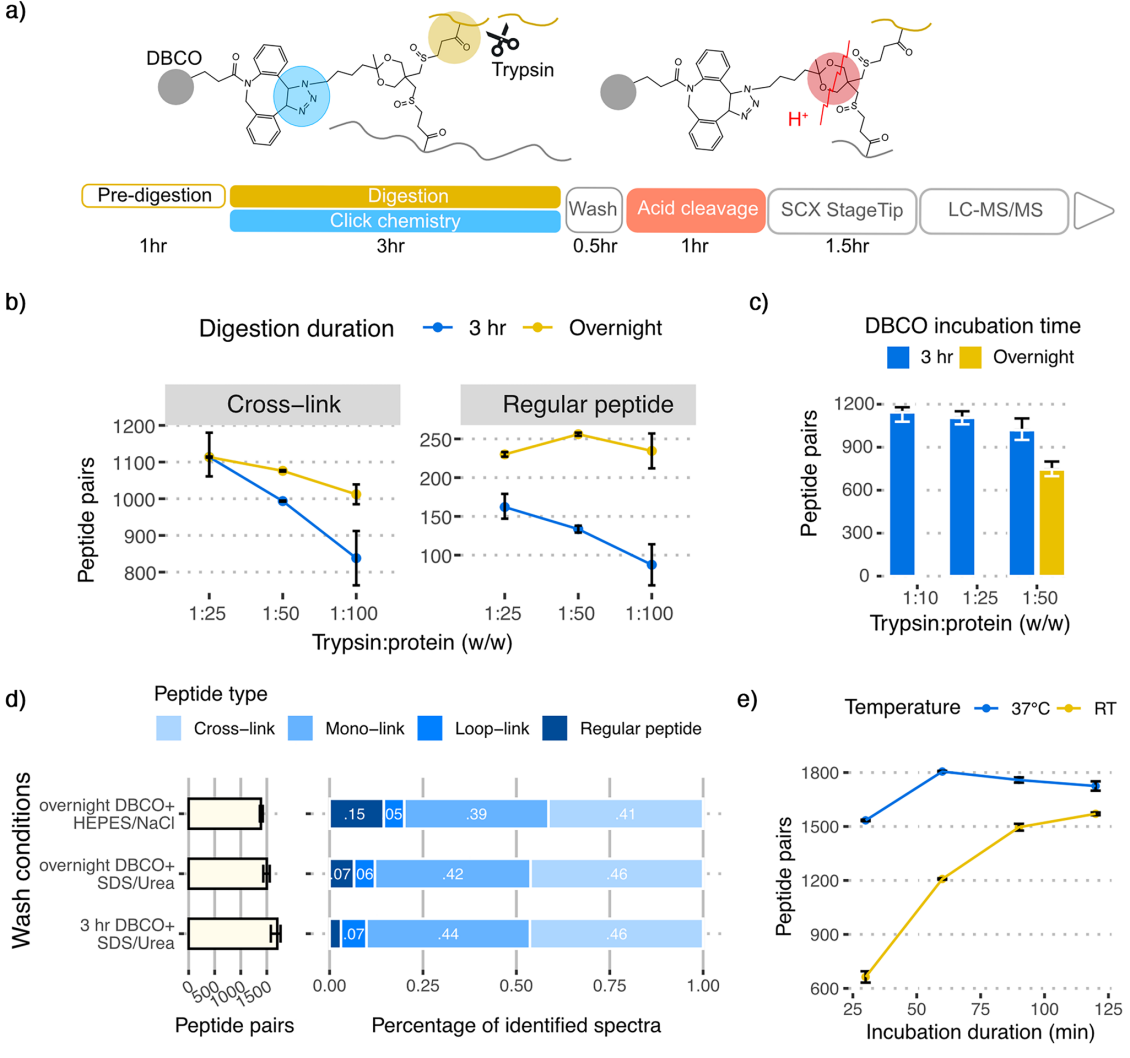
Optimization of protein digestion and cross‐link enrichment workflow using DSBSO cross‐linked HEK293T cells. a) Schematic timeline of enzymatic digestion and DBCO‐azide enrichment b) Evaluation of cross‐link identification across different trypsin amounts and digestion times. c) Comparison of cross‐link identification across different trypsin amounts and varying incubation times of DBCO agarose beads. d) Assessment of cross‐link identification number and enrichment specificity under different washing buffer conditions and bead incubation durations. e) Evaluation of acidic cleavage efficiency using 2% TFA at different temperatures and incubation times. *Data points represent the mean of technical replicates (*n* = 2 for b, e; *n* = 3 for c, d). Error bars show the range (from min to max).*

We cross‐linked intact HEK293T cells with azide‐A‐DSBSO to generate a complex biological sample for optimizing the sample preparation and MS data acquisition protocol. After cross‐linking, published DSBSO workflows require two overnight reactions to perform digestion, click chemistry‐based bead coupling, and acidic elution.^[^ ^11^, ^25^, ^26^ ^]^ We shortened this process to 5 h by integrating trypsin digestion with DSBSO‐DBCO bead coupling (Figure 1a).

We performed an initial digestion with trypsin and Lys‐C for one hour to release heavily cross‐linked proteins into medium‐sized peptides. Subsequently, we added DBCO beads to initiate the click chemistry reaction. During incubation with DBCO beads, trypsin continues to digest medium‐sized peptides to fully trypsinized peptides. We systematically optimized trypsin amount and incubation time (Figure 1b). We identified most cross‐links (reported at the peptide‐pair level throughput the manuscript unless otherwise stated) after a 3 h digestion at a protein‐to‐enzyme ratio of 1:25, increasing unique cross‐link identifications by 40% compared to overnight incubation (Figure 1c,d, and Figure S1).

Next, we benchmarked strategies to wash off nonspecifically bound linear peptides and subsequently release the cross‐linked peptides. The most efficient published DSBSO workflow from Matzinger et al. used a HEPES‐NaCl buffer,^[^ ^25^ ^]^ but we found SDS‐urea from the original DSBSO paper to be the optimal washing solution (Figure 1d). For release of the cross‐links, the Matzinger DSBSO workflow includes a 1 h incubation in 2% trifluoroacetic acid (TFA) at room temperature. We find that a 1 h incubation at 37 °C increases cross‐link identifications by 50% (Figure 1e).

### Improving Sample Desalting and Contaminant Removal

**Figure 2:**
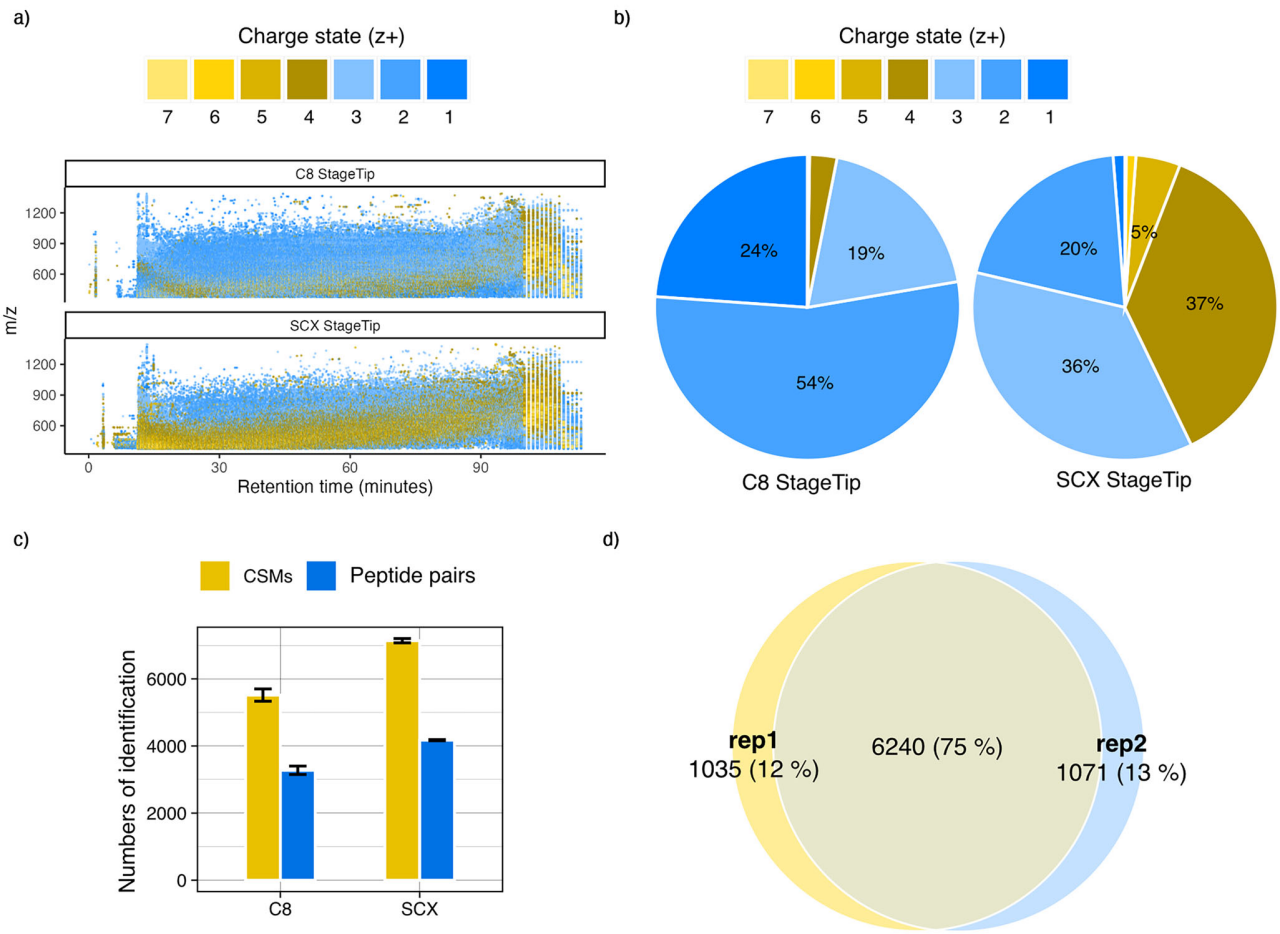
Performance comparison of C8 and SCX StageTip methods for XL‐MS sample clean‐up using DSBSO cross‐linked HEK293T cells. a) Charge state distribution of MS1 features across the entire chromatogram in C8 and SCX StageTip samples. b) Percentage of different charge states of all detected MS1 features. c) Number of identifications of C8 desalting and SCX StageTip. d) Overlap of cross‐link identifications between two technical replicates (two injections of the same sample). *Data points represent the mean of technical replicates (*n* = 2 for c, d). Error bars show the range (from min to max).*

The standard approach for removing non‐volatile salts from cross‐linked peptide mixtures prior to LC‐MS is reversed‐phase separation using C8 StageTips or columns.^[^ ^8^ ^]^ When we analyzed C8‐desalted samples following cross‐link enrichment, we observed a substantial population of + 1/+2‐charged species in the LC chromatogram throughout the entire gradient (Figure 2a and Figure S2), contributing 78% of the MS1 features (Figure 2b). We hypothesize that these species are non‐peptide contaminants that eluted from the DBCO agarose beads upon acid treatment and were not removed by C8 desalting. Supporting this hypothesis, drying the DBCO bead eluate yielded gel‐like aggregates with aggregation increasing at higher acid concentrations (Figure S3).

To eliminate these interfering species, we replaced C8 separation by a single‐step SCX StageTip‐based elution using a volatile, MS‐compatible 500 mM ammonium acetate solution. Moving from C8 to SCX StageTip reduced the MS1 signals of + 1/+2‐charged species from 78% to 21% (Figures 2a,b) and increased cross‐link identifications by 32% (Figure 2c). Replicate analysis demonstrated a 75% overlap in cross‐link identifications, confirming the robustness of our DSBSO enrichment and SCX clean‐up protocol (Figure 2d).

### Fine‐Tuning of Gas‐Phase Separation and Fragmentation

**Figure 3:**
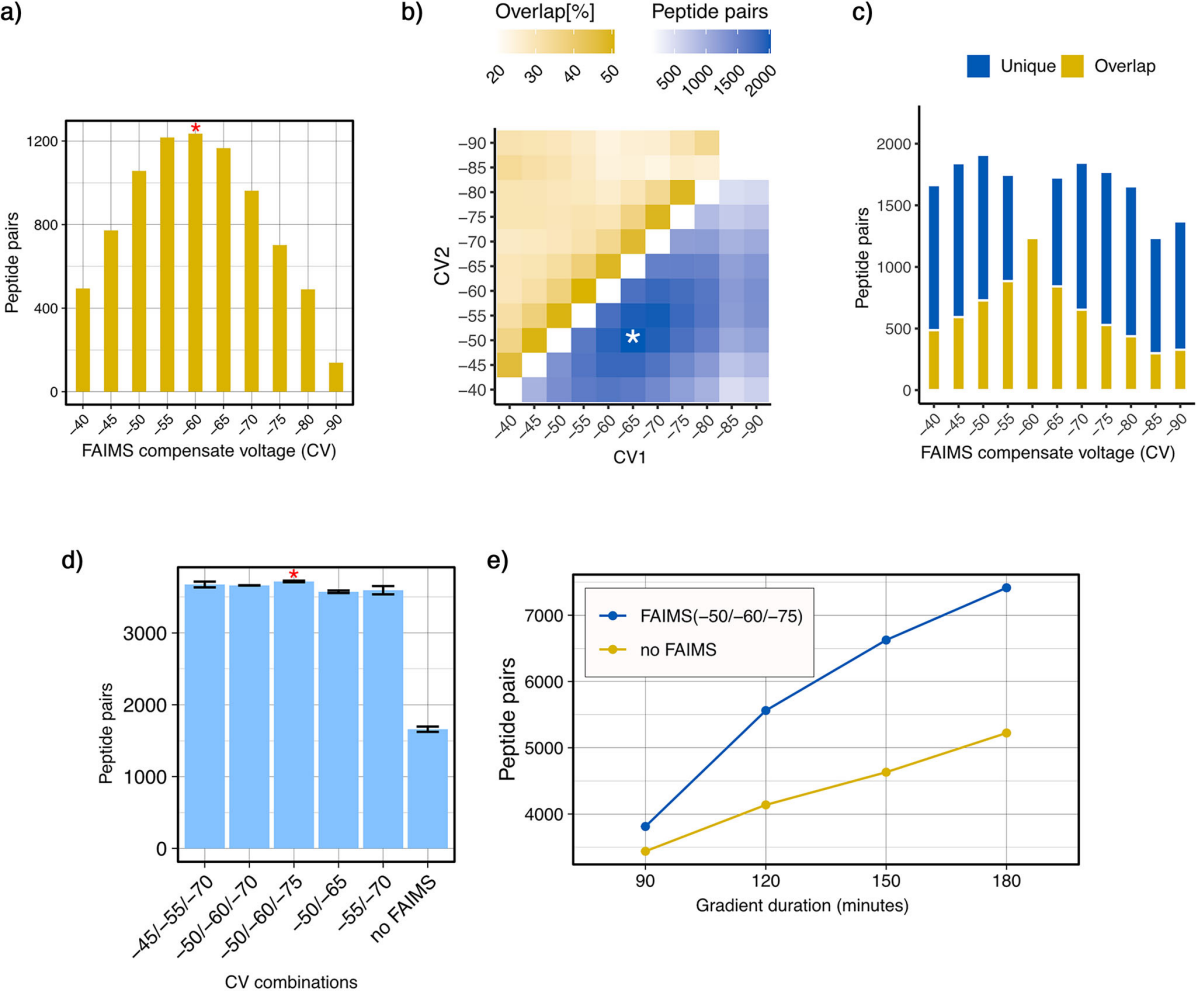
Optimization of FAIMS CV settings for DSBSO‐cross‐linked peptides. a) Evaluation of individual CV values based on CSMs and unique cross‐link identifications using Scout. b) Pairwise CV combinations were evaluated based on the overlap of unique cross‐links. The asterisk marks the most complementary CV pairing. c) Determination of optimal triple‐CV setting by overlapping each candidate CV with –60 (the top‐performing single CV from panel A). d) Number of identifications with FAIMS of different CV combinations and without FAIMS in a 2hr gradient. Data points represent the mean of technical replicates (*n* = 2). Error bars show the range (from min to max). e) Number of identifications with and without FAIMS at different gradient lengths.

Having streamlined the XL‐MS sample preparation in solution, we next focused on the gas‐phase separation and fragmentation of DSBSO‐cross‐linked peptide ions in the MS. We have previously shown that separating peptide ions by FAIMS improves cross‐link identification.^[^ ^27^ ^]^ Pursuing a similar strategy, we determined individual FAIMS compensation voltages (CVs) and 2‐CV combinations that maximize the unique cross‐link identifications (Figure 3a–c), indicating that CVs should be between −45 and −75. FAIMS 3‐CV combinations in this range show additional minor improvements over 2‐CV combinations and −50/−60/−75 was optimal in our hands for DSBSO‐based XL‐MS (Figure 3d). FAIMS doubled cross‐link identifications compared to non‐FAIMS methods in a 2h gradient (Figure 3d) with greater improvements observed in longer gradients (Figure 3e).

**Figure 4:**
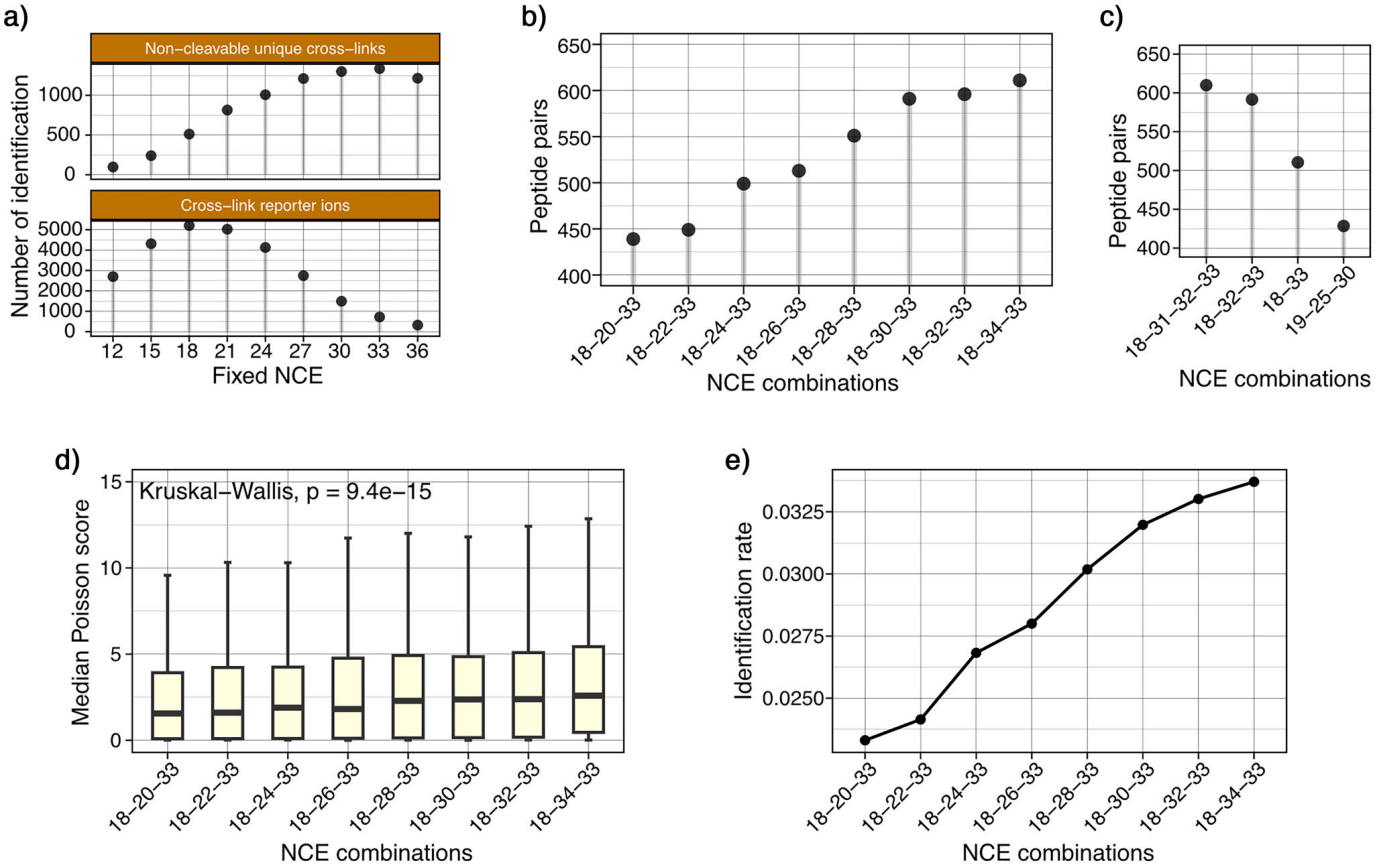
Optimization of HCD energies for cross‐link identification. a) Evaluation of cross‐link identification across single HCD normalized collision energies (NCEs), using pLink3 software in non‐cleavable cross‐linker search mode (top), or using XlinkX software to count the numbers of cross‐link reporter ions (bottom). b) Optimization of middle HCD NCE (20–34, increments of 2) with fixed low (18) and high (33) NCEs using Scout. c) Comparison of cross‐link identification of selected stepped HCD NCE combinations. d) Distribution of cross‐link Poisson scores ^[^ ^24^, ^28^ ^]^ at different stepped HCD settings. Poisson scores were determined by Scout and higher scores indicate better spectral quality. The Kruskal‐Wallis test revealed a significant trend of increasing median Poisson scores with higher middle HCD NCE. e) Evaluation of identification rate (number of cross‐link spectrum matches (CSMs) divided by number of MS2 events) at different stepped HCD settings.

Finally, we sought to identify HCD settings for the optimal generation of fragment ions from peptide backbone cleavage as well as reporter ions from DSBSO cleavage. To this end, we first conducted LC‐MS measurements using varying single HCD normalized collision energies (NCEs) and evaluated two Parameters: 1) the number of cross‐links detected by pLink3 (v3.0.17) (Mao et al., manuscript in preparation), which disregards DSBSO reporter ions and 2) the number of reporter ion signals identified by XlinkX3.0. We observed optimal peptide fragmentation at a HCD‐NCE of 33 and optimal reporter ion generation at a HCD‐NCE of 18 (Figure 4a). Combining Different HCD‐NCEs in a stepped HCD experiment was shown to be beneficial when working with cleavable cross‐linkers.^[^ ^29^, ^30^ ^]^ Therefore, We fixed HCD‐NCEs between 18 and 33 while scanning for an optimal third NCE Step, which was found in the range of 30–34 (Figure 4b). Using a 4^th^ NCE step in the same range gives similar results (Figure 4c). The low‐high‐high and low‐high‐high‐high HCD‐NCE combinations improve peptide fragmentation (shown by Poisson scoring of the cross‐links^[^ ^24^, ^28^ ^]^) and Identification Rates (Figure 4d,e). By Contrast, Low‐medium‐high HCD‐NCE Combinations, which were recommended in earlier DSBSO protocols,^[^ ^29^ ^]^ reduce cross‐link identifications by 10%‐20% (Figure 4b).

### SCX Stage Tip‐based Fractionation for *Bacillus Subtilis* Interactome Analysis

**Figure 5:**
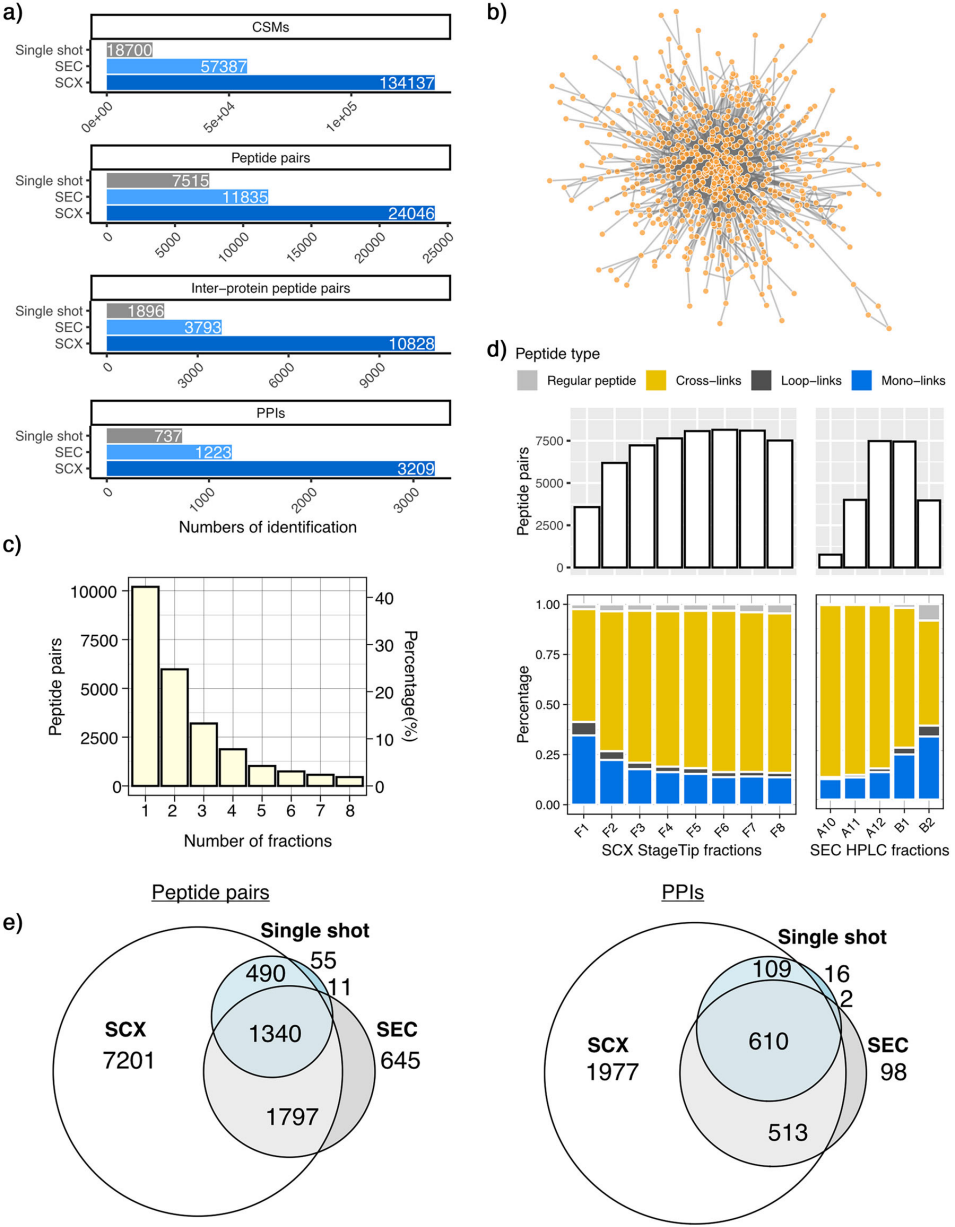
XL‐MS based interactome of *Bacillus subtilis*. a) Number of identifications in single‐shot measurement, SEC fractionated or SCX fractionated samples. b) Interactome of *B. subtilis* containing 24046 unique cross‐linked peptides and 3209 PPIs. c) Number of cross‐links identified in each SCX fraction. d) Percentage of different digestion products across all SCX fractions. e) Overlap of identifications from measurements of single‐shot, SEC fractions, and SCX fractions.

After optimizing our DSBSO XL‐MS workflow on HEK cell samples, we wanted to assess its general applicability on a complex sample. Therefore, we cross‐linked intact *Bacillus subtilis*, which contains a proteome of 4191 reviewed protein sequences in UniProt. We prepared the sample as described above and performed a single shot LC‐MS measurement after StageTip‐SCX. This yielded 737 PPIs at 1% peptide pair‐FDR from 1 µg input of enriched cross‐links (derived from 100 µg of digested peptides prior to DBCO enrichment) (Figure 5a). For comparison, a published *B. subtilis* PPI network generated using the non‐enrichable, cleavable DSSO cross‐linker and extensive chromatographic cross‐link enrichment by SCX and SEC identified 560 PPIs at 2.5% PPI‐FDR from 2400 µg of peptide input.^[^ ^31^ ^]^ The efficiency of our workflow becomes even more evident when considering instrument time: we achieved competitive identification numbers in a single 3 h gradient run, compared to the 228 instrument hours needed in the prior study.

Previous work has shown that, even when enrichable cross‐linkers like DSBSO or PhoX are applied, detection sensitivity benefits from separating the low abundant cross‐linked peptides from the excess of linear peptides by chromatographic approaches such as SEC.^[^ ^11^, ^12^ ^]^ Since SEC as a column‐based method requires substantially more input sample than our streamlined protocol, we looked for an alternative that can enhance cross‐link detection depth without compromising the efficiency of our workflow. Therefore, we expanded the single‐step SCX‐StageTip clean‐up to an eight‐step fractionation with increasing ammonium acetate concentrations (40–500 mM). Combining all fractions yielded 24046 unique cross‐linked peptides, corresponding to 3209 PPIs, representing a comprehensive *B. subtilis* interactome (Figures 5a,b). We found that 67% of cross‐links were identification in 1–2 fractions, indicating good separation between adjacent SCX fractions (Figure 5c).

For direct benchmarking of our StageTip‐based SCX workflow, we subjected the same cross‐linked *B. subtilis* sample to SEC and collected five early fractions for LC‐MS analysis. The SEC workflow required 15 times more input sample than StageTip‐SCX (120 µg vs. 8 µg). Despite its high sample consumption, the SEC workflow gave less than half as many identifications as the SCX workflow on CSM, cross‐link, residue pair, and PPI level (Figure 5a). Within individual fractions, SCX and SEC showed similar ability to enrich cross‐links over other species (mono‐links, loop‐links, and linear peptides), indicating the improvement of SCX over SEC is primarily attributable to superior separation between fractions (Figure 5d). Accordingly, the unique cross‐link identifications were distributed more evenly across SCX fractions compared to SEC fractions. In SEC, only two fractions achieved identification levels comparable to SCX fractions (Figure 5d), confirming previous protocols that analyze 2–5 SEC fractions for enrichable cross‐linkers.^[^ ^11^, ^12^ ^]^ The vast majority of cross‐links identified in the SEC workflow as well as the single‐shot measurement were also found with the SCX fractionation protocol, further confirming that SCX can replace SEC (Figure 5e). Finally, Gene Ontology (GO) analyses confirmed that the additional PPIs identified through SCX fractionation expand coverage across diverse Biological Process (GOBP) and Cellular Component (GOCC) categories (Figure S4). Furthermore, the XL‐MS‐derived PPIs are enriched for high‐confidence STRING scores, validating the high quality of the resulting interactome (Figure S5).

## Conclusion

Enrichable cross‐linkers hold great promise for improving detection sensitivity in XL‐MS studies. In this work, we substantially enhanced the sensitivity, efficiency, and throughput of proteome‐wide XL‐MS using the enrichable cross‐linker azide‐A‐DSBSO. By optimizing the workflow, we reduced the protocol duration from several days to just 10 h while achieving a 4‐fold increase in cross‐link identifications in single‐shot measurements. A key factor in this improved sensitivity was the effective removal of interfering + 1/+2‐charged contaminant ions, which significantly enhanced spectral quality.

Additionally, we implemented StageTip‐based SCX fractionation, which dramatically improved cross‐link detection depth while requiring minimal sample input. In *Bacillus subtilis*, this approach yielded a 2‐fold increase in cross‐link identifications alongside a 15‐fold reduction in sample consumption compared to conventional SEC fractionation.

The efficiency of our SCX‐StageTip workflow is further underscored by a recent study that also employed DSBSO in a customized workflow.^[^ ^32^ ^]^ While this work reported ∼5000 cross‐links from in vivo cross‐linking of human cells (K562), our approach identified 24046 cross‐links from *Bacillus subtilis* using the same instrument hours (12 fractions × 2 h in their study vs. 8 fractions × 3 h in our study) and similar amount of starting material (∼1 mg protein per replicate in both studies).

In summary, we present a proteome‐wide XL‐MS protocol that requires only a fraction of the time and resources consumed by published state‐of‐the‐art approaches, while affording similar detection sensitivity. This efficiency and scalability make our XL‐MS workflow primed for interactome studies in high‐throughput and on low‐input samples.

## Author Contributions

P.J. Designed and performed experiments; analyzed data; and created figures. Y.Z., J.C., M.W., and C.W.: Assisted the experiments. F.L.: Supervised the research and wrote the manuscript. All authors revised the manuscript.

## Conflict of Interests

F.L. is a shareholder and advisory board member of Absea Biotechnology and VantAI. The remaining authors declare no Conflict of interests.

## Supporting information



Supporting Information

## Data Availability

The data that support the findings of this study are openly available in [PRIDE] at [https://www.ebi.ac.uk/pride/], reference number [PXD065905].
