# Supplementary material for: Optimized In‐Solution and Gas‐Phase Chemistry Enables High‐Efficiency Interactome Mapping by DSBSO‐Based Cross‐Linking Mass Spectrometry
Source: Angew Chem Int Ed Engl. 2025 Nov 20;65(4):e18355. doi: 10.1002/anie.202518355 (PMC12828450; doi:10.1002/anie.202518355)
Supplement: Supplementary file 1 — Supporting Information [file ANIE-65-e18355-s001.docx]

**Optimized In-Solution and Gas-Phase Chemistry Enables High-Efficiency Interactome Mapping by DSBSO-Based Cross-Linking Mass Spectrometry**

Pin-Lian Jiang^1,2^, Ying Zhu^1,3^, Jiaxin Cai^1,3^, Cong Wang^1^, Mei Wu^1^, Ke Pu^1,2^, Fan Liu^*,1,4^

1. Leibniz-Forschungsinstitut für Molekulare Pharmakologie (FMP), Berlin, Germany

2. Freie Universität Berlin, Germany

3. Absea Biotechnology Ltd, Berlin, Germany

4. Charité Universitätsmedizin Berlin, Germany

# Contents:

1. Materials and Methods

2. Supplementary Figure 1

3. Supplementary Figure 2

4. Supplementary Figure 3

5. Supplementary Figure 4

6. Supplementary Figure 5

7. Supplementary Table 1

**Material and Methods**

Cross-linking of HEK293T cells and *Bacillus subtilis*

HEK293T cells were harvested and washed three times with PBS. The sample concentration was determined using Bradford assay and adjusted to a final concentration of 10 mg/mL with PBS. azide-A-DSBSO powder was dissolved in pure DMSO to prepare a 50 mM stock solution. The cross-linker was then added to the sample to achieve a final concentration of 4 mM. Cross-linking reaction was carried out at room temperature for 15 minutes on a rotating mixer or shaker. The reaction was quenched by adding 20 mM Tris-HCl (Carl Roth), followed by an additional 15-minute incubation with shaking. The sample was centrifuged, and the supernatant was discarded. The pellet was washed again with PBS to remove unreacted DSBSO. After washing, the pellet was either stored at –20 °C or processed further.

*Bacillus subtilis* was revived from glycerol stocks by inoculation into LB medium and incubated at 37 °C with continuous shaking at 180 rpm. After 24 hours of growth, the culture reached mid-log phase with an optical density at 600 nm (OD₆₀₀) of approximately 0.6. Cells were harvested by centrifugation at 3,800 g for 10 minutes at 4 °C and subsequently washed three times with ice-cold PBS. Protein concentration was determined using the BCA protein assay kit following the manufacturer's instructions. The cell pellet was resuspended in PBS to a final protein concentration of 10 mg/mL and cross-linked with 2 mM azide-A-DSBSO for 15 min at room temperature with gentle shaking. The reaction was quenched with 20 mM Tris-HCl (pH 8.0) for 30 min at room temperature under the same mixing conditions. Cross-linked cells were pelleted again by centrifugation at 3,800 g for 10 min at 4 °C From the total cross-linked *Bacillus subtilis* proteins, 200 µg was allocated for SCX-StageTip single elution, 800 µg for SCX-StageTip fractionation, and 4 mg for SEC for further sample processing.

Cell lysis and protein digestion

The sample was resuspended in 8 M urea (Carl ROTH) to adjust the protein concentration to < 5 mg/mL. Cell lysis was performed by sonication (30 sec on/30 sec off cycles for 5 min in total at 4 °C). The lysate was centrifuged at 13,000 × g for 20 minutes at 4 °C, and the supernatant was collected for further processing. Protein concentration was determined using the bicinchoninic acid assay (BCA assay, Thermo Fisher Scientific). Disulfide bonds were reduced with 5 mM dithiothreitol (DTT, Avantor Sciences) at 37 °C on a shaker for 30 minutes, followed by alkylation of cysteine residues using 50 mM 2-chloroacetamide (CAA, Sigma-Aldrich) in 50 mM tetraethylammonium bromide (TEAB, pH 8.0, Sigma-Aldrich) for an additional 30 minutes at 37 °C. After diluting Urea to 2M with 50 mM TEAB, proteolysis was initiated by adding trypsin at a 1:25 (w/w) enzyme-to-substrate ratio and Lysyl endopeptidase C (Wako) at a 1:50 (w/w) ratio simultaneously. The digestion was carried out for 1 hour at 37 °C on a shaker.

DBCO agarose beads conjugation and cleaning

During the 1-hour digestion, a slurry of dibenzocyclooctyne-coated agarose beads (DBCO agarose beads, Vector Laboratories) was prepared at a sample-to-slurry ratio of 100:15 (w/v). The beads were washed three times with anhydrous acetonitrile (ACN, Fisher chemical), using at least 5-fold the bead volume for each wash. The beads were then resuspended in ACN and added to the sample immediately after the 1-hour digestion. The digestion and click reaction were continued together for an additional 3 hours at 37 °C on a shaker. After incubation, the sample was centrifuged at 2,000 × g for 1 minute, and the supernatant was discarded. The conjugated beads were then incubated in 0.5% sodium dodecyl sulfate (SDS, Carl ROTH) in water at 37 °C on a shaker for 15 minutes. Following incubation, the sample was centrifuged and transferred to a spin column placed on a waste collection tube. All subsequent washing steps using the spin column were performed at 2,000 × g for 1 minute. The SDS solution was first eluted by centrifugation. The beads were then washed three additional times with 0.5% SDS. Subsequently, the sample was washed three times with 8 M urea in water, followed by three washes with water. For each washing step, the volume of buffer used was at least five times the volume of the DBCO beads.

Acid cleavage and elution

After washing, the plug of the spin column was re-locked, and five volumes (relative to the bead volume) of 2% trifluoroacetic acid (TFA, Sigma-Aldrich) were added for acid cleavage. The spin column with new tube was capped and incubated on a shaker at 37 °C for 1 hour. After acid cleavage was completed, the entire spin column with tube, including the cap and plug, was centrifuged at 300 × g for 1 minute. The plug was then removed, followed by opening the cap of the spin column. The sample was eluted by centrifugation at 2000 × g for 1 minute. For the second elution, the same volume used in the first elution was applied using 80% ACN in 0.1% trifluoroacetic acid (TFA). For the third elution, deionized water was used at twice the volume of the first elution.

Sample cleaning using SCX StageTip

A single layer of SCX membrane, cut from a commercial SCX disk (CDS analytical), was embedded into a 200 μL pipette tip (greiner) using a 16-gauge syringe. The prepared StageTip was mounted on a waste collection tube using an adaptor. The SCX membrane was first activated with 20 μL of methanol (Fisher chemical), followed by 20 μL of 500 mM ammonium acetate (NH₄OAc, Merck) in 0.5% acetic acid (AA)/20% acetonitrile (ACN). Each step was centrifuged at 1000 × g for 1 minute. Then membrane was then washed twice with 20 μL of 0.5% AA/20% ACN under the same centrifugation conditions. The sample eluted from the spin column was directly loaded onto the StageTip at 1000 × g. Peptides bound to the SCX membrane were then washed twice with 20 μL of 50 mM NH₄OAc in 0.5% AA/20% ACN. For elution, the StageTip was placed into a clean tube, and bound peptides were eluted twice using 20 μL of 500 mM NH₄OAc in 0.5% AA/20% ACN at 1000 × g. The eluted buffer containing NH₄OAc was evaporated using a SpeedVac at 45 °C for 30 minutes. The dried sample was then resuspended in 100 μL of deionized water, briefly centrifuged, and dried again at 45 °C in the SpeedVac (Thermo Fisher Scientific). The final dried sample was stored at –20 °C or subjected to LC-MS/MS analysis.

Size-exclusion chromatography (SEC) fractionation

The dried sample from DBCO elution was resuspended in 30% acetonitrile (ACN) in deionized water to achieve a final concentration below 4 μg/μL. Fractionation was performed using a Thermo Fisher Vanquish Flex UHPLC system equipped with a Superdex™ 30 Increase 3.2/300 column (GE Healthcare). A total of 24 fractions were collected over a 60-minute gradient at a flow rate of 0.005 mL/min. The first nine early eluting fractions were completely dried using a SpeedVac and subjected to subsequent LC-MS/MS analysis.

LC-MS analysis

DSBSO-cross-linked samples were resuspended in 0.1% formic acid (FA, Buffer A, Avantor Sciences) and analyzed using either an Orbitrap Fusion Lumos system (Thermo Fisher Scientific) coupled with an UltiMate 3000 RSLC nano LC system (Thermo Fisher Scientific), or an Orbitrap Exploris 480 system (Thermo Fisher Scientific) coupled with a Vanquish Neo LC system system (Thermo Fisher Scientific). Peptides were separated using reversed-phase chromatography on an in-house packed C18 analytical column (Poroshell 120 EC-C18, 2.7 μm, Agilent Technologies). For the 160 minutes LC gradient (excluding washing and equilibration), Buffer B (100% acetonitrile in 0.1% trifluoroacetic acid, Avantor Sciences) was ramped up from 4% to 8% over the first 3 minutes, and increased linearly to 20% over the next 102 minutes. From 105 to 158 minute, buffer B was raised to 32%, followed by 5-minute column wash with 80% Buffer B and a 10-minute re-equilibration in 100% buffer B. The 2-hour gradient was proportionally shortened based on the 3-hour gradient profile. FAIMS Pro interface (Thermo Fisher Scientific) was installed in front of the ion source and operated with internal compensation voltage (CV) stepping at –50, –60, and –75 V. A spray voltage of 2200 V was applied. MS1 scans were acquired in the Orbitrap with a resolution of 120,000 over an m/z range of 375–1600 in positive ion mode, using a standard AGC target and a maximum injection time of 50 ms. Precursor ions were selected for MS/MS if their intensity exceeded 2.0 × 10⁴ and charge states ranged from +4 to +6. Dynamic exclusion was enabled with a 60-second exclusion window for previously fragmented precursors. The instrument operated in Top Speed mode with a 2-second cycle time. MS2 scans were acquired using stepped higher-energy collisional dissociation (stepped HCD) with normalized collision energies of 18, 31, 32, and 33. Fragment ions were analyzed in the Orbitrap at a resolution of 50,000 with a maximum injection time of 86 ms, a normalized AGC target of 200%, and an isolation window of 1.6 m/z. In proteomic measurements, digested non-cross-linked peptides were desalted using C18 Sep-Pak (Waters) cartridges. Samples were analyzed by LC-MS/MS using a 3-hour reversed-phase gradient. Data-dependent acquisition (DDA) was performed with a full scan range of m/z 375–1200. Fragmentation was conducted using fixed higher-energy collisional dissociation (HCD) with a normalized collision energy of 30. MS2 spectra were acquired in the Orbitrap at a resolution of 15,000.

Cross-link identification

Cross-link search was performed using a reduced database (Human: 6,640 sequences, Bacillus subtilis:2,353 sequences). The reduced database was generated from proteins identifications obtained by searching non-cross-linked peptide data against reviewed databases from Homo sapiens database (UniProt; 20,301 sequences) or Bacillus subtilis (UniProt; 4,191 sequences). Cross-linked samples were searched against the reduced database using either pLink 3.0.17 (https://github.com/pFindStudio/pLink3)or Scout 1.5.1 (https://github.com/theliulab/Scout) with the following parameters: enzyme specificity, trypsin; three missed cleavages; fixed medication, Cysteine carbamidomethylation (+57.0215 Da); variable modification, methionine oxidation (+15.9949 Da); the DSBSO mass, 308.039 Da; long arm, 236.018 Da; short arm, 54.011 Da; cross-linking site, lysine side chain and protein N-termini. For pLink3, the results were filtered at 1% false discovery rate (FDR) at the peptide pair level. For Scout, 1% FDR was set for all levels (i.e., CSM, residue-pair and PPI). Results from pLink3 were exported at the CSM level and identifications were aggregated to residue pair and PPI levels. Results from Scout were exported directly at the CSM, residue-pair and PPI levels. Figure 4D is processed by Scout 2.0.0 beta with the same parameters as stated above (manuscript in preparation).

Statistics and Data Sources

Samples were randomly allocated to experimental groups and replicates. Number of replicates for each experiment is summarized in Supplementary Table 1. Data points represent the mean of technical replicates. Error bars show the range (min to max). A Kruskal–Wallis test was applied in Figure 4d to assess the trend significance of Poisson scores derived from Figure 4b. Bacillus DSBSO samples for SCX and SEC (Figure 5) were cross-linked simultaneously and then processed separately. The image in Supplementary Figure 3 was acquired using a Nikon D750 with a 105 mm macro lens.

**
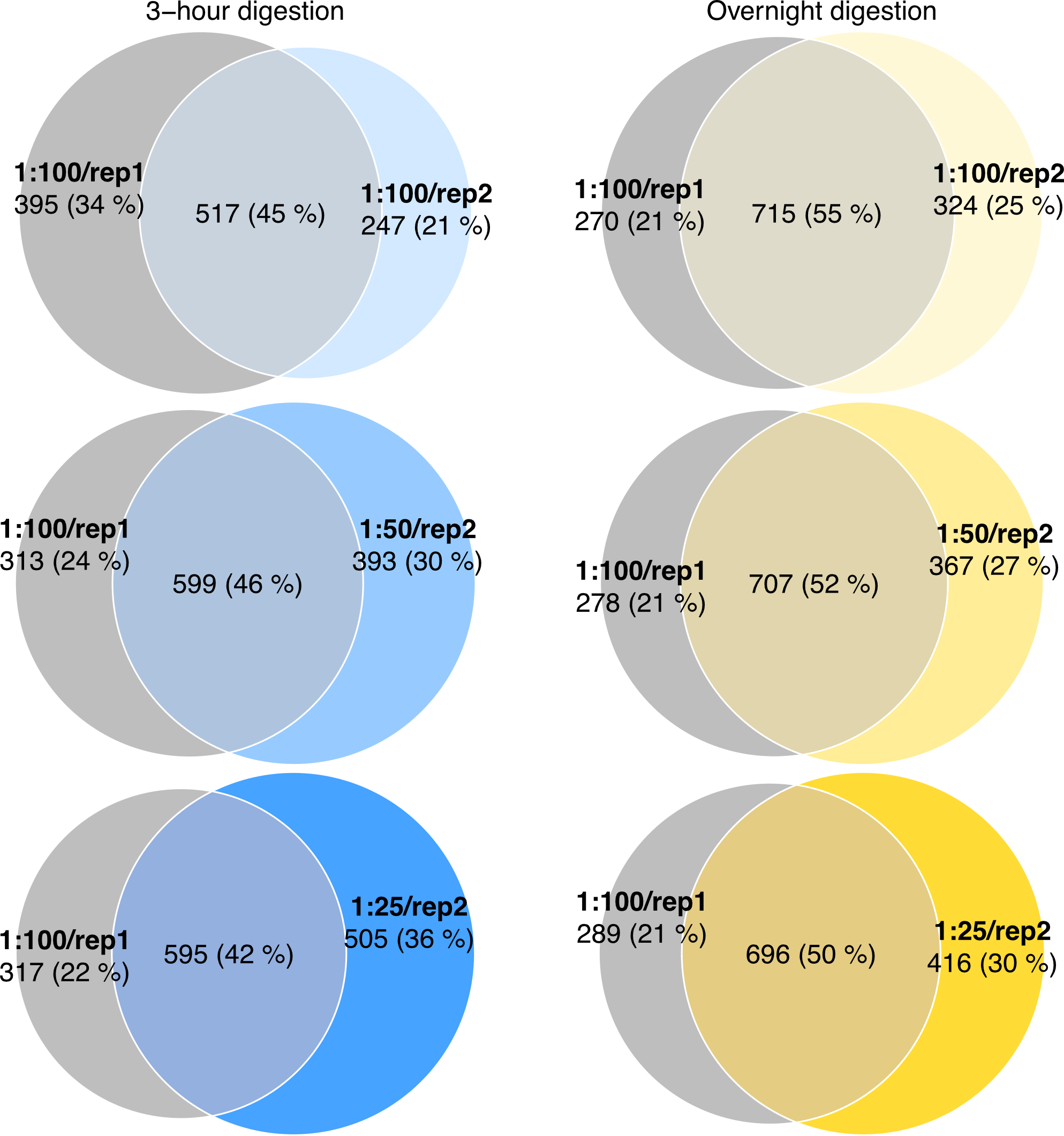
**

**Supplementary Figure 1. Overlap of cross-links between replicates and between experiements using different trypsin amounts.** For both 3-hour (left) and overnight digestions (right), increasing trypsin concentration resulted in more cross-links while maintaining an overlap comparable to that between replicates.

**
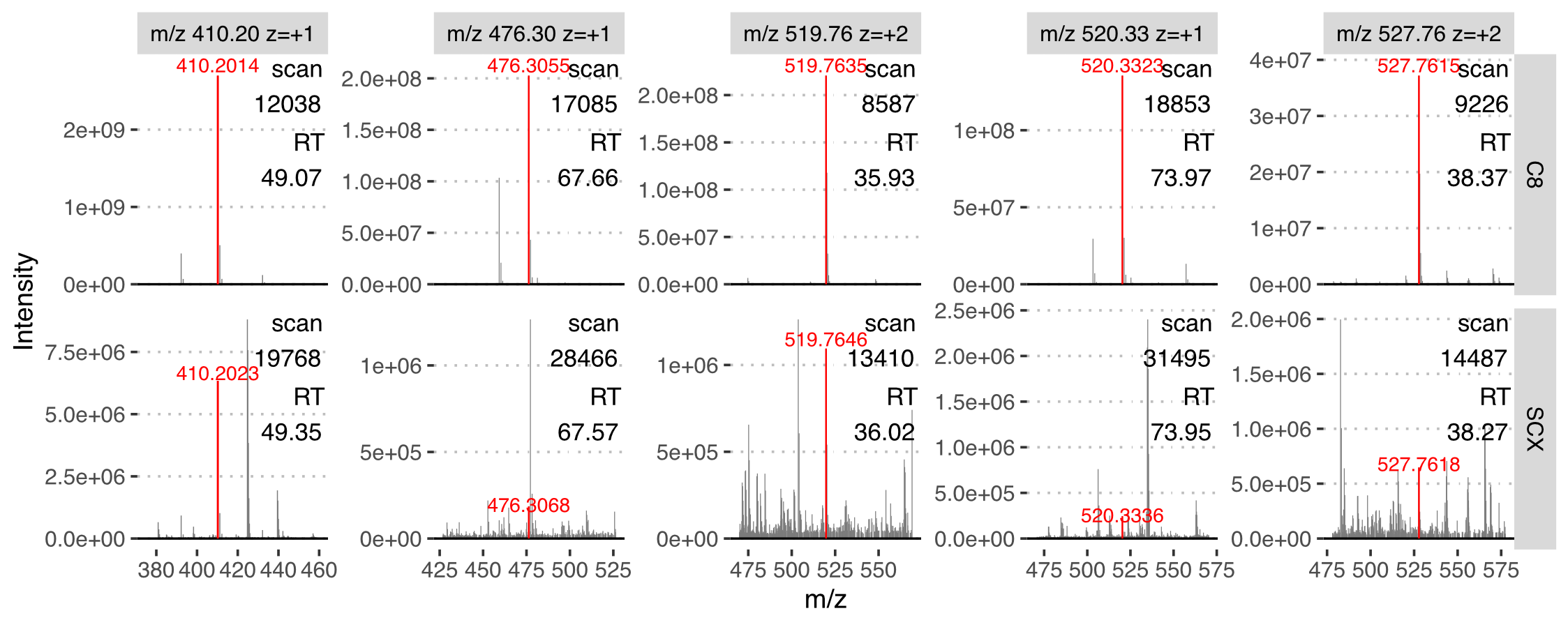
**

**Supplementary Figure 2. Reduction of contaminants by SCX StageTip.** Representative MS1 spectra showing +1/+2 charged contamination peaks in cross-link enriched samples using either C8 desalting (upper panel) or SCX StageTip (lower panel). Significant reduction of low-charge-state contaminants was observed through SCX StageTip clean-up.


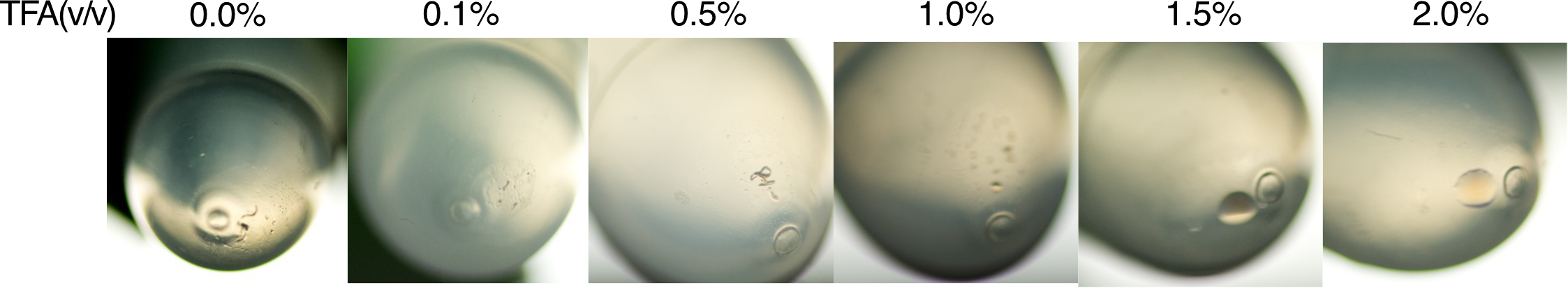


**Supplementary Figure 3. Acid concentration-dependent aggregation in DBCO bead eluates.** Formation of gel-like aggregates observed upon drying of eluates from DBCO bead enrichment. Amount of aggregation increases with higher acid concentrations during cross-linker cleavage.


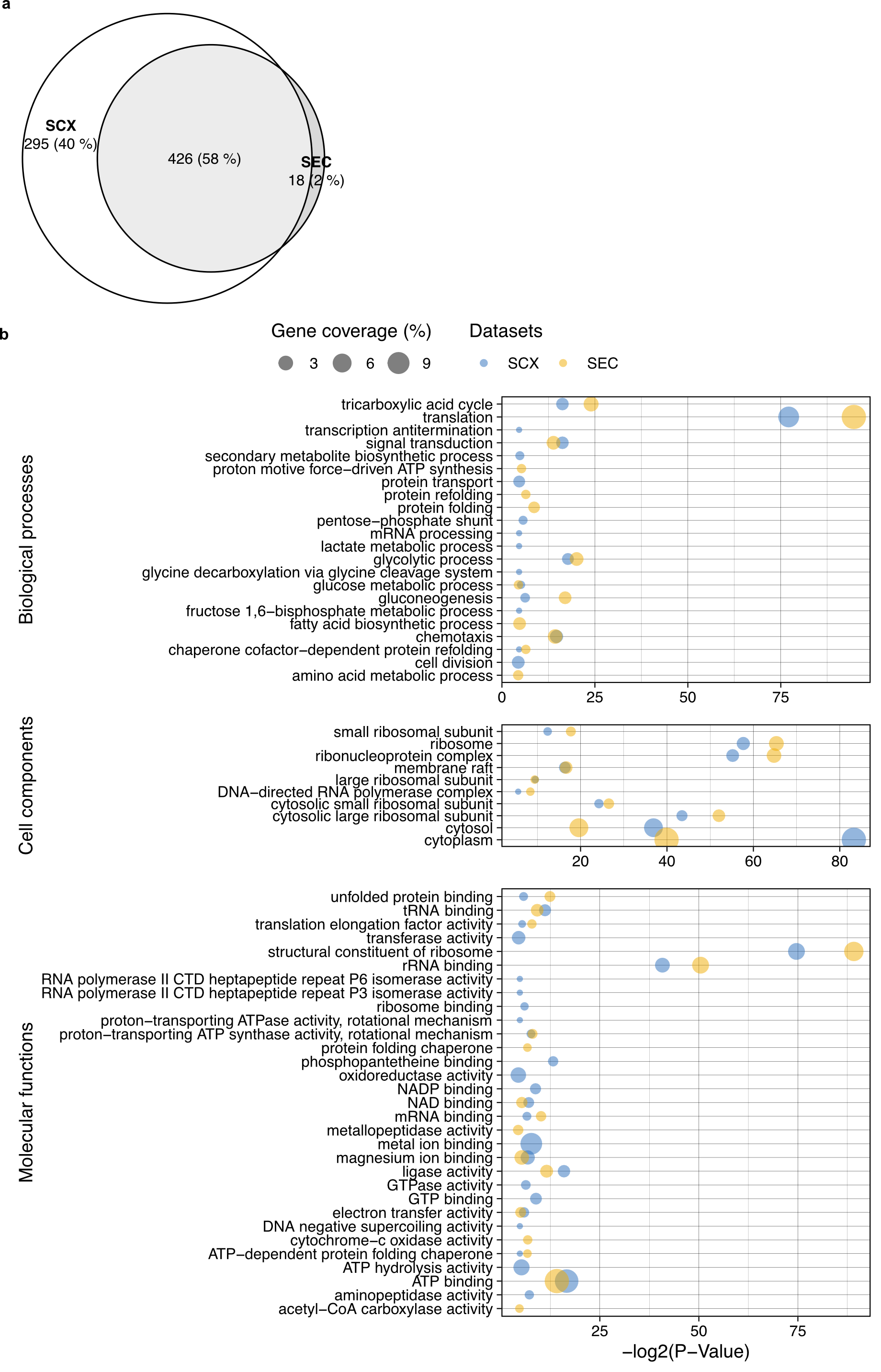


**Supplementary Figure 4.** Functional enrichment analysis of proteins involved in cross-links. **(a)** Overlap of cross-linked proteins using SCX and SEC methods. **(b)** GO enrichment analysis for cellular components, biological processes, and molecular functions. Enrichment was performed using DAVID (*p* < 0.05).


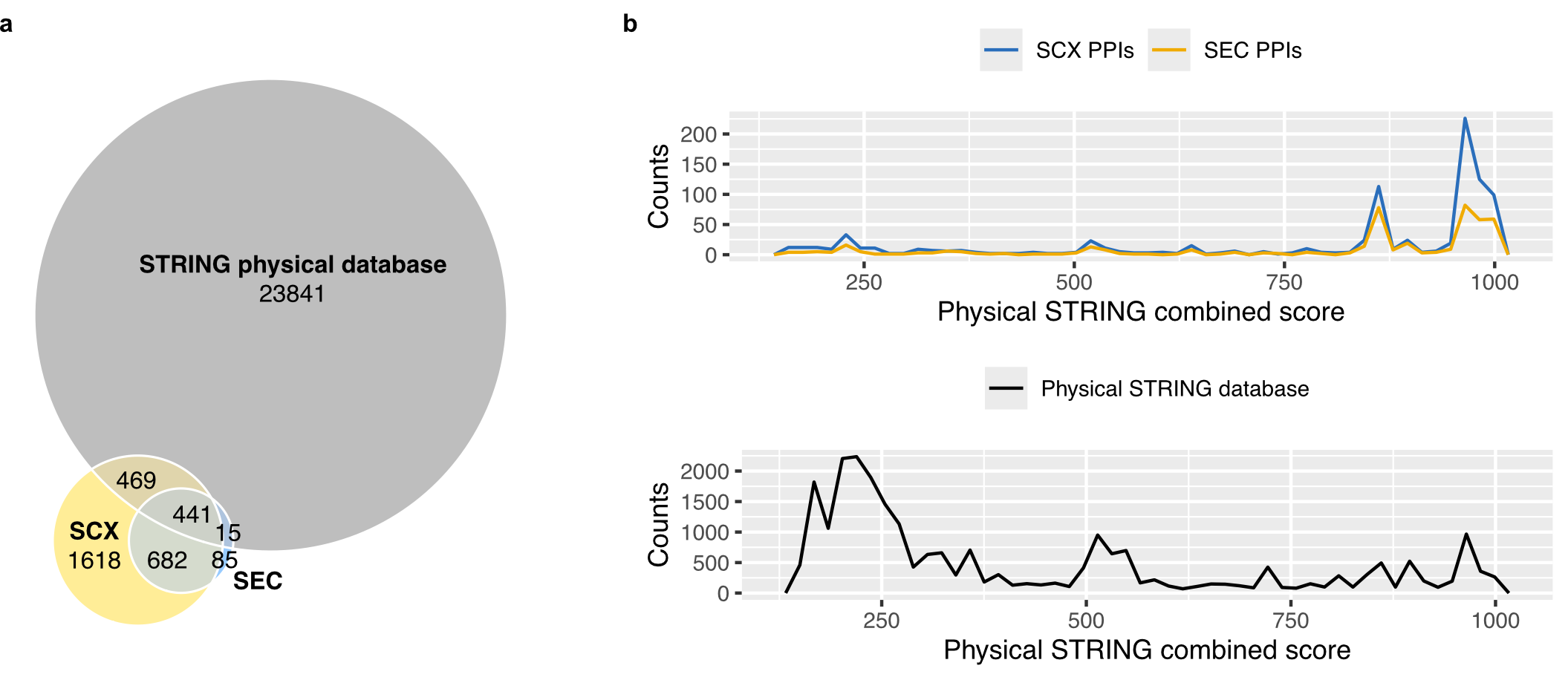


**Supplementary Figure 5.** **Comparison of XL-MS-derived PPIs with PPIs reported in the STRING database.** **(a)** Overlap of PPIs identified by SCX, SEC and experimentally supported physical interactions in the STRING database. **(b)** Distribution of STRING combined confidence scores for PPIs from the SEC dataset, SCX dataset, and the STRING database.

| Figure | Software | FDR level | FDR | Replicate |
| --- | --- | --- | --- | --- |
| 1b | Scout1.5.1 | All | 1% | 2 |
| 1c | Scout1.5.1 | All | 1% | 3 |
| 1d | Scout1.5.1 | All | 1% | 3 |
| 1e | Scout1.5.1 | All | 1% | 2 |
| 2a | Maxquant 2.6.3.0 | None | None | 1 |
| 2b | Maxquant 2.6.3.0 | None | None | 1 |
| 2c | pLink3.0.17 | Peptide pair | 1% | 2 |
| 2d | pLink3.0.17 | Peptide pair | 1% | 2 |
| 3a | Scout1.5.1 | All | 1% | 1 |
| 3b | Scout1.5.1 | All | 1% | 1 |
| 3c | Scout1.5.1 | All | 1% | 1 |
| 3d | pLink3.0.17 | Peptide pair | 1% | 2 |
| 3e | pLink3.0.17 | Peptide pair | 1% | 1 |
| 4a (upper) | pLink3.0.17 conventional mode | Peptide pair | 1% | 1 |
| 4a (lower) | XlinkX 3.0 | None | None | 1 |
| 4b | Scout1.5.1 | All | 1% | 1 |
| 4c | Scout1.5.1 | All | 1% | 1 |
| 4d | Scout2.0.0 beta | All | 1% | 1 |
| 4e | Scout1.5.1 | All | 1% | 1 |
| 5a | pLink3.0.17 | Peptide pair | 1% | 1 |
| 5b | pLink3.0.17 | Peptide pair | 1% | 1 |
| 5c | pLink3.0.17 | Peptide pair | 1% | 1 |
| 5d | pLink3.0.17 | Peptide pair | 1% | 1 |
| 5e | pLink3.0.17 | Peptide pair | 1% | 1 |

**Supplementary Table 1. Summary of key experimental parameters and search engines used in this study.** The table details the software, FDR cutoff and the number of replicates for each experiment.
